# Sevoflurane postconditioning ameliorates cerebral ischemia-reperfusion injury in rats via TLR4/MyD88/TRAF6 signaling pathway

**DOI:** 10.18632/aging.204461

**Published:** 2022-12-29

**Authors:** Zijun Zhao, Yishuai Li, Fei Chi, Li Ma, Yanan Li, Zhiyong Hou, Qiujun Wang

**Affiliations:** 1 Department of Anesthesiology, The Third Hospital of Hebei Medical University, Shijiazhuang 050051, Hebei, China; 2 Department of Anesthesiology, Hebei Provincial Chest Hospital, Shijiazhuang 050047, Hebei, China; 3 Department of Thoracic Surgery, Hebei Provincial Chest Hospital, Shijiazhuang 050047, Hebei, China; 4 Department of Oncology, Hebei Provincial Chest Hospital, Shijiazhuang 050047, Hebei, China; 5 Surgical Department of Clinical Medicine, Shijiazhuang People’s Medical College, Shijiazhuang 050091, Hebei, China; 6 Department of Orthopaedics, The Third Hospital of Hebei Medical University, Shijiazhuang 050051, Hebei, China

**Keywords:** sevoflurane postconditioning, TLR4/MyD88/TRAF6 signaling pathway, erebral ischemia-reperfusion, neuroinflammation, bioinformatics analysis

## Abstract

To determine whether sevoflurane postconditioning protects against cerebral ischemia reperfusion (I/R) injury and its potential mechanism, we employed bioinformatic analysis, neurological assessments, and western blot analysis, as well as triphenyl tetrazolium chloride, hematoxylin and eosin, Nissl, and immunofluorescence staining. We identified 103 differentially expressed genes induced by cerebral I/R, including 75 upregulated genes and 28 downregulated genes enriched for certain biological processes (involving regulation of inflammatory responses, cellular responses to interleukin 1, and chemokine activity) and signaling pathways (such as transcriptional misregulation in cancer, interleukin-17 signaling, rheumatoid arthritis, MAPK signaling, and Toll-like receptor signaling). As a typical path in Toll-like receptor signaling pathway, in the current study, we investigated the protective effect of sevoflurane postconditioning in cerebral I/R rats and further explore the role of TLR4/MyD88/TRAF6 signaling pathway in it. The results showed cerebral I/R-induced neurological deficits were comparatively less severe following sevoflurane postconditioning. In addition, TLR4/MyD88/TRAF6 signaling pathway-related proteins and neuropathic damage were ameliorated in aged rats following sevoflurane postconditioning, while the TLR4 agonist lipopolysaccharide aggravated these changes. Together, these findings suggest that sevoflurane postconditioning ameliorates cerebral I/R injury by a mechanism involving inhibition of the TLR4/MyD88/TRAF6 signaling pathway to suppress neuroinflammatory responses.

## INTRODUCTION

With an increasing aging population, significant increases in cardiovascular and cerebrovascular diseases have been observed [1, 2]. Worldwide, stroke is currently the second most common cause of death [3]. Moreover, strokes have become one of the most common neurological conditions in recent years [4]. About 85% of strokes in China are ischemic strokes, which are among the most lethal and disabling diseases [5]. Cerebral ischemia reperfusion (I/R) injury occurs after blood flow is restored, aggravating ischemia-induced damage to tissues and organs [6, 7]. Thus, finding therapeutics for cerebral I/R and understanding their protective mechanism is a major challenge for researchers.

Although the mechanism of cerebral I/R is not entirely clear, numerous studies have confirmed that cell apoptosis [8], blood-brain barrier destruction [9], increased neuroinflammatory responses [7], neuronal cytosolic calcium overload [10], and oxidative stress [11] are closely associated with the occurrence of cerebral I/R. Researchers have demonstrated that inflammation is crucial to the onset and progression of cerebral I/R. Moreover, cerebral I/R can induce overexpression of cyclooxygenase in vascular endothelial cells, which will destroy the balance of thromboxane A2 and prostaglandin 2, increase neuroinflammatory responses, and cause angiogenic cerebral edema and neuronal damage [12]. Evidence demonstrates that acute brain injury can be caused by cerebral I/R and activation of microglia [13], macrophages [14], and monocytes [15]. These findings suggest that inflammation contributes to tissue damage after brain I/R, although the underlying mechanisms are unknown.

Sevoflurane is commonly used as an inhalation anesthetic in the clinic. Sevoflurane postconditioning has increasingly been studied in recent years for its neuroprotective effects [16–18]. In several studies, postconditioning with sevoflurane was demonstrated to protect against cerebral I/R-induced damage. A study by Shi et al. [19] found that sevoflurane postconditioning protected rats from cerebral I/R-induced brain injury by inhibiting autophagy and apoptosis. In addition, sevoflurane postconditioning has been shown to inhibit lysosomal cathepsin B activation and release after ischemic stroke, and attenuate astrogliosis and glial scar formation [20]. In cerebral I/R rats, sevoflurane postconditioning decreased blood and brain oxidative injury to enhance immunity indexes [21]. Cellular inflammatory and immunological responses are significantly influenced by the Toll-like receptor 4 (TLR4)/MyD88/tumor necrosis factor receptor-associated factor 6 (TRAF6) signaling pathway. Indeed, chronic inflammation and autoimmune diseases may be caused by perturbed regulation of the TLR4/MyD88/TRAF6 pathway [22]. Zhang et al. demonstrated that high mobility group box 1 (HMGB1)-mediated changes in HMGD4/MyD88/TRAF6 expression attenuated cerebral I/R injury through knockdown of pyruvate kinase isozymes M1/M2, providing a potential target for treating cerebral I/R injury [23]. However, the role of sevoflurane postconditioning in protection against cerebral I/R remains unclear.

Current evidence suggests that there is an intimate connection among neuroinflammatory responses, the TLR4/MyD88/TRAF6 signaling pathway, and sevoflurane postconditioning in cerebral I/R rats. Therefore, the purpose of this study was to determine whether sevoflurane postconditioning protects rats against cerebral I/R injury and explore the underlying mechanisms. Future clinical research will be guided by the results of this study.

## MATERIALS AND METHODS

The Chinese Guidelines for the Care and Use of Laboratory Animals were followed for all experimental animals. Throughout the study, every effort was made to minimize animal suffering and use as few animals as possible. The Animal Review Board of the Third Hospital of Hebei Medical University approved all protocols pertaining to animals (Ethical code: 2021-005-1).

### Bioinformatics analysis

We downloaded the gene expression dataset for cerebral I/R (GSE23160) from the Gene Expression Omnibus database (http://www.ncbi.nlm.nih.gov/gds/) and used the R language Limma package (version 3.40.6) to analyze gene expression differences. R was used to perform principal component analysis, and ggplot2 was used to generate a volcano chart of differentially expressed genes (DEGs). The R software package pheatmap was used to create a heatmap of DEG clusters.

### Analyses of functional enrichment

Analysis of gene ontology (GO) was conducted by integrating GO terms and networks, while DEGs for biological processes were created using the Database for Annotation, Visualization, and Integrated Discovery (DAVID) (http://david.ncifcrf.gov/). The Limma package (|logFC| < 1, p < 0.05) was used to obtain common DEGs, which were analyzed for enrichment by GO and Kyoto Encyclopedia of Genes and Genomes (KEGG) analyses. GO terms and networks were integrated using DAVID online database tools to analyze the level of biological processes for DEGs. R was used to draw GO pathway maps of DEGs. Enrichment analysis of all genes was conducted using Gene Set Enrichment Analysis (GSEA), and pathways were mapped on the basis of GSEA [24, 25].

### Experimental animals and group assignments

We purchased 120 healthy male Sprague-Dawley rats from the Experimental Animal Center of Hebei Medical University (Permit No. SCXK 2021-004). Rats were aged 3 months and weighed 280–350 g each. Rats were maintained in a controlled environment with a temperature range of 26–28° C, 40%–60% humidity, and 12-h light/dark cycle. Using computer-based randomization, rats were divided into four groups after 1 week of adaptive feeding (n = 30 per group): control (Group C), cerebral I/R (Group IR), cerebral I/R plus sevoflurane (Group IR+S), and cerebral I/R plus sevoflurane and TLR4 agonist (Group A).

**Figure 1:**
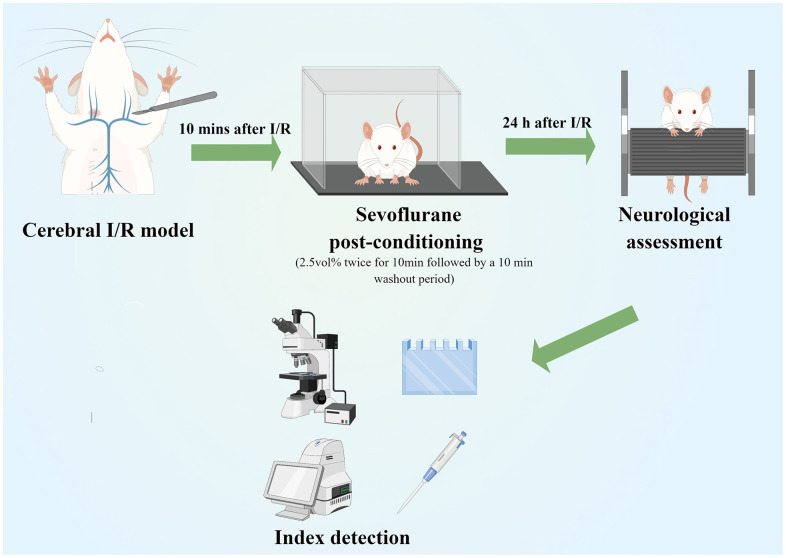
**Experimental flow chart of the study (Drawn by Figdraw platform, ID:TRWIW954c9).** A cerebral I/R model was established by middle cerebral artery occlusion. Sevoflurane postconditioning was performed 10 min after reperfusion. Neurological assessment was established at 24 h after reperfusion. Afterwards, rats were sacrificed for western blot analysis, as well as TTC, HE, Nissl and immunofluorescence staining.

In accordance with a previous study, rats in IR+S and A groups were twice administered 2.5 vol% sevoflurane by inhalation for 10 min, followed by a 10 min washout period after ischemia I/R [26]. Immediately following reperfusion, rats in Group A were intraperitoneally (i.p.) administered 50 μg/kg of the TLR4 agonist lipopolysaccharide (LPS; Sigma-Aldrich, St. Louis, MO, USA) [27, 28]. A similar amount of normal saline was injected through the tail veins of the other three groups (Figure 1).

### Establishment of cerebral I/R model

The cerebral I/R model was established in IR, IR+S, and A group rats using middle cerebral artery occlusion (MCAO) [8]. First, anesthesia was administered i.p. with sodium pentobarbital (40 mg/kg). On the right side of the head, the common carotid artery, external carotid artery, and internal carotid artery were exposed through an incision in the midline. In the external carotid artery, a small incision was made at the bifurcation of the common carotid artery. Nylon fishing line (0.26-mm diameter; Ethicon, Raritan, NJ) was inserted into the external carotid artery lumen for 18–20 mm until a slight resistance was felt. Two hours after the nylon fishing line was removed, rats were returned to their cages for reperfusion.

### Neurological deficit scores

Twenty-four hours after reperfusion, a blinded investigator assessed the neurological status of rats in accordance with a previously described protocol for neurological scoring [29]. According to this protocol, rats scored 0 points when they behaved normally, 1 point when they were unable to fully extend their left front legs, 2 points when they turned around in circles, 3 points when they fell on their left side, and 4 points when they were unable to move independently and lost consciousness.

### 2, 3, 5-triphenyltetrazolium chloride (TTC) staining

Following neurological status assessment, five rats from each group were sacrificed after deep anesthesia with sodium pentobarbital (60 mg/kg, i.p.) and their left-brain tissues were collected. After washing brains with physiological saline for 5 min, 1-mm thick slices were cut using a brain slice mold. Next, fresh brain sections were soaked in 2% TTC in phosphate buffered saline (PBS) for 20 min in darkness at room temperature. Subsequently, sections were washed with PBS and fixed for 30 min at room temperature in 4% paraformaldehyde (Sigma-Aldrich). Brain sections were photographed using a digital camera, and the infarct area (unstained area) was measured with ImageJ 1.51 software (http://www.imagej.nih.gov/ij). The following formula was used to calculate the infarct volume: (infarct area/total brain area) × 100%.

### Hematoxylin and eosin (HE) staining

After neurological status assessment, left brain tissues from rats were collected (n = 5) and then immersed in 4% paraformaldehyde for 24 h. After dehydration and embedment of the samples in paraffin wax, the paraffin-embedded 5 μm coronal brain sections containing the hippocampus were sliced and HE-stained. The pathological changes in the hippocampus were examined using optical microscopy.

### Nissl staining

The left-brain tissues from rats (n = 5) were collected and fixed in 4% paraformaldehyde after neurological status assessment. Paraffin-embedded coronal brain sections containing the hippocampus were obtained after dehydrating the samples and embedding them in paraffin wax. After being sequentially submerged in xylene and a graded alcohol series to remove the paraffin, sections were dyed for 20 min at room temperature with 1% cresyl violet. Next, sections were separated with 70% alcohol, submerged and rinsed once in distilled water, and then submerged for 2 min each in a graded alcohol series, followed by xylene. Finally, sections were mounted on neutral balsam and examined using an optical microscope, and images were acquired with a camera.

### Enzyme-linked immunosorbent assay (ELISA)

Following neurological assessment, five rats from each group were decapitated and their brain tissue was dissected. As described above, protein samples were extracted and protein concentrations were determined. Each group of duplicate standards and samples was analyzed to determine the mean absorbance. Commercial ELISA kits (R&D Systems, Minneapolis, MN) were used to measure levels of tumor necrosis factor (TNF-α), interleukin-1β (IL-1β), and interleukin-6 (IL-6) in brain tissue in accordance with the manufacturer’s protocols. A 1-pg/mL sample of total protein was used to measure cytokine levels.

### Immunofluorescence assay

After neurological status assessment, five rats from each group were sacrificed and their left brains were collected and rinsed with PBS. Sections of the hippocampus (20-mm thick) were sliced for immunofluorescence staining and blocked for 2 h at room temperature with PBS containing 5% bovine serum album (Beyotime, Beijing, China) and 0.3% Triton X-100 (Sigma-Aldrich). Subsequently, sections were incubated overnight at 4° C with an anti-TLR4 antibody (1:100; ab22048; Abcam, Cambridge, UK), anti-MyD88 antibody (1:100; ab133739, Abcam), anti-TRAF6 antibody (1:100; ab40675, Abcam) or anti-Neun antibody (1:100; ab177487, Abcam). After three rinses with PBS, a secondary goat anti-rabbit IgG-FITC antibody (P0186, Beyotime) was applied to sections and incubated for 1 h at room temperature. Finally, after staining sections with 4ʹ,6-diamidino-2-phenylindole (DAPI; P0131, Beyotime) for 10 min, images were captured using a Nikon Eclipse CI fluorescence microscope and analyzed using Image Pro Plus 6.0 (Media Cybernetics, Rockville, MD).

### Western blotting

Radioimmunoprecipitation buffer containing a protein phosphatase inhibitor (Sigma-Aldrich) was used to lyse the left hippocampal tissues of rats from each group (n = 5). After extracting the total proteins, they were transferred to a polyvinylidene fluoride membrane, blocked for 2 h, and incubated with primary antibodies (1:1,000; Abcam) against TLR4 (ab217274), MyD88 (ab133739), and TRAF6 (ab33915), followed by appropriate secondary antibodies. To visualize blots, an ultra-sensitive FujiFilm LAS 4000 chemiluminescent liquid-based imaging analyzer was used. The relative intensities of individual bands were analyzed using ImageJ.

### Statistical analysis

Statistical analysis was conducted using SPSS 21.0 (SPSS, Chicago, IL). Data are expressed as mean ± standard deviation (SD). For data with a normal distribution, a one-way analysis of variance with Tukey’s multiple comparison test was used to evaluate differences. For non-normal distributions, Kruskal-Wallis and Dunn-Bonferroni tests were used to assess differences. Statistical significance was determined by a P value of 0.05 or less.

### Data availability statement

The data generated during and/or analyzed during the current study are available from the corresponding author on reasonable request.

## RESULTS

### Screening of DEGs

**Figure 2:**
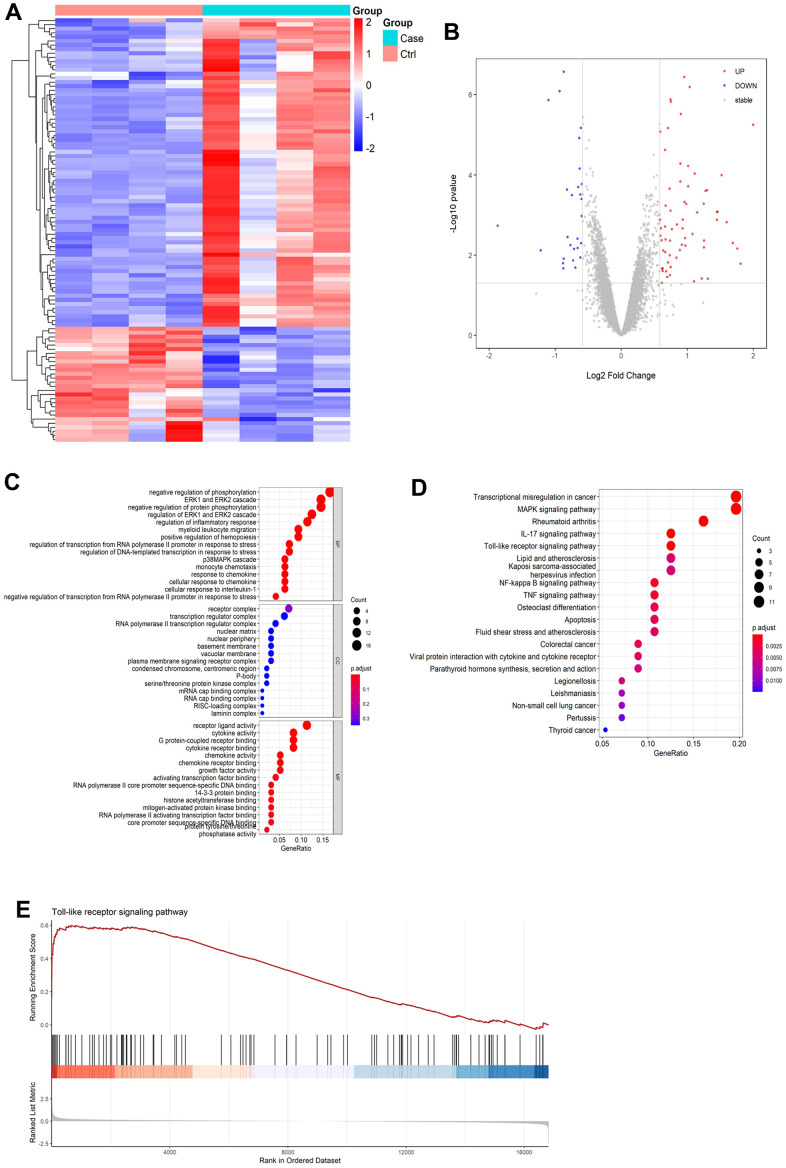
**Results of bioinformatics analysis.** (**A**) Differential gene volcano map. (**B**) Heatmap of differentially expressed gene cluster analysis. (**C**) GO enrichment analysis pathway diagram. (**D**) KEGG enrichment analysis pathway diagram. (**E**) GSEA gene enrichment analysis diagram of Toll-like receptor signaling pathways.

We performed bioinformatics analysis to analyze gene expression differences in cerebral I/R model. As shown in Figure 2A, a volcano map of DEGs in the GSE23160 dataset was constructed. In addition, a DEG cluster analysis heatmap was created (Figure 2B). The results revealed 103 DEGs, of which 75 were upregulated and 28 were downregulated (Supplementary Table 1).

### Results of the bioinformatics analysis

Functional enrichment analysis of DEGs were performed by GO and KEGG enrichment analyses. In accordance with the GO pathway diagram, DEGs were enriched for “signal release from regulation of inflammatory response,” “cellular response to interleukin-1,” and “chemokine activity” (Figure 2C and Supplementary Table 2). KEGG pathway analysis indicated significant enrichment of 162 signaling pathways, including 89 for upregulated DEGs and 73 for downregulated DEGs (Supplementary Table 3). According to the KEGG pathway map, “transcriptional misregulation in cancer,” “IL-17 signaling pathway,” “rheumatoid arthritis,” “MAPK signaling pathway,” and “Toll-like receptor signaling pathway” were the top five enriched pathways (Figure 2D). Additionally, GSEA revealed significant enrichment of Toll-like receptor signaling pathways (enrichment score:0.54, *P*=0.001, Figure 2E and Supplementary Figure 2).

### Sevoflurane postconditioning ameliorated neurological scores and infarction volumes after cerebral I/R injury

**Figure 3:**
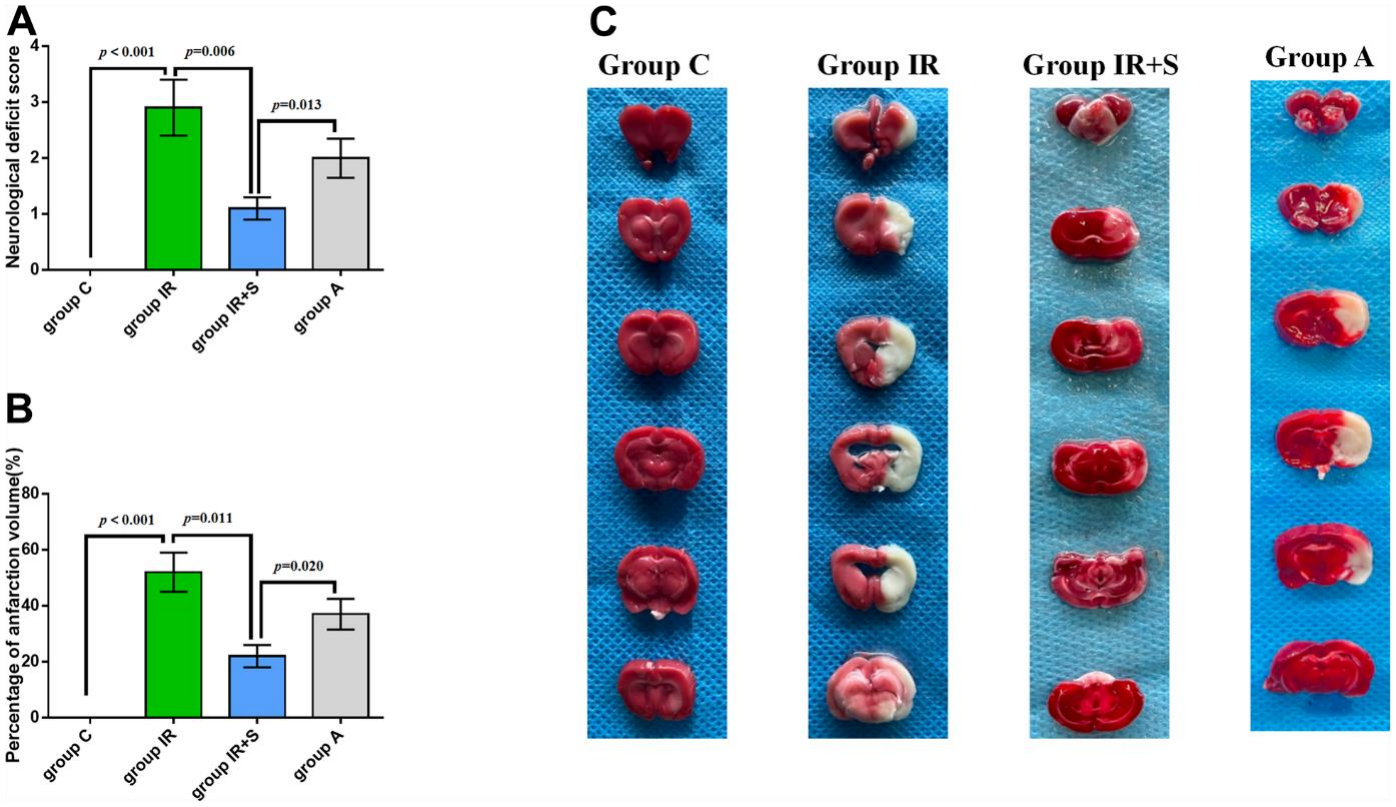
**Sevoflurane postconditioning ameliorated neurological scores and infarction volumes after cerebral I/R injury.** (**A**) Neurological deficit score of each group (n = 30 per group). (**B**) Percentage of infarction volume of each group (n = 5 per group). (**C**) Representative sections of TTC staining in each group. Data are shown as means ± SD.

Following cerebral I/R, infarct volumes and neurological scores were assessed to determine whether sevoflurane postconditioning impacted brain ischemia. As shown in Figure 3A–3C, Group IR showed higher neurological deficit scores (*p* < 0.001) and focal infarction volumes(*p* < 0.001) compared with Group C, consistent with previous research [30, 31]. However, sevoflurane postconditioning reversed the increase of neurological deficit scores (*p* = 0.006) and focal infarction volumes (*p* = 0.011) induced by cerebral I/R in Group IR+S. Furthermore, Group A had higher neurological deficit scores (*p* = 0.013) and focal infarction volumes (*p* = 0.020) than Group IR+S.

### Sevoflurane postconditioning reduced neuropathic damage after cerebral I/R injury

**Figure 4:**
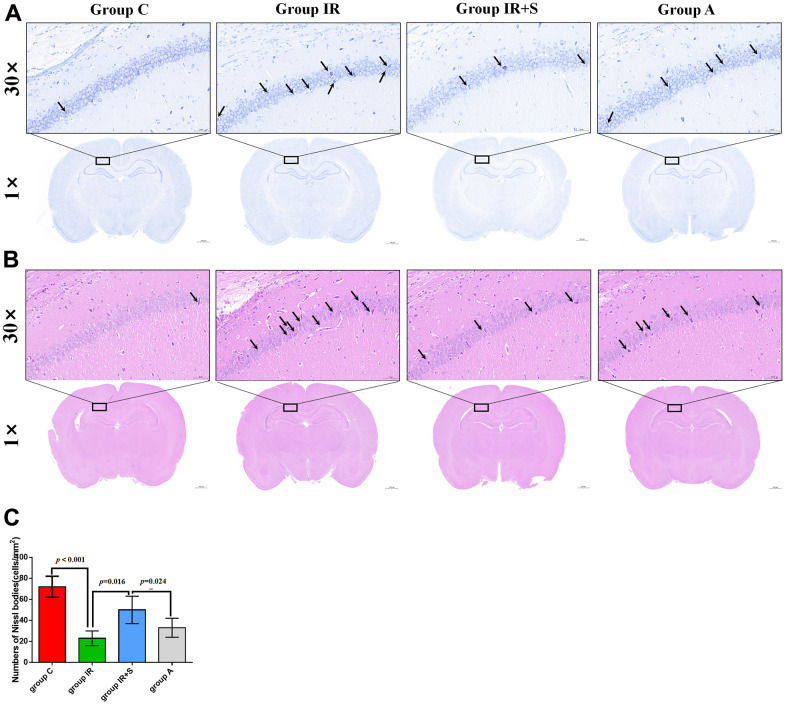
**Sevoflurane postconditioning reduced neuropathic damage after cerebral I/R injury.** (**A**) Representative photomicrographs of Nissl staining in the hippocampal CA1 region (scale bar = 50 μm and 1000 μm respectively). The arrow points to shrunk and degenerated cells in CA1 region. (**B**) Representative images of histopathological changes in the hippocampal CA1 of rats (scale bar = 50 μm and 1000 μm respectively). The arrow points to shrunk and degenerated cells in CA1 region. (**C**) Quantification of numbers of Nissl bodies in the hippocampal CA1 region. Data are expressed as mean ± SD (n = 5 per group).

Using Nissl and HE staining, we further examined the protective effect of sevoflurane postconditioning on neuropathic damage in cerebral I/R rats. Histological analysis revealed no structural abnormalities in the CA1 region in Group C, and the neurons were aligned. Rats in Group IR, however, displayed significantly reduced Nissl bodies (*p* < 0.001) and disorganized neurons after cerebral I/R. Sevoflurane postconditioning significantly increased Nissl bodies (*p* = 0.016) and restored normal alignment and structures in the CA1 area in Group IR+S compared with Group IR. The TLR4/MyD88/TRAF6 pathway agonist LPS could partially reverse the protective effect of sevoflurane postconditioning in Group A (*p* = 0.024, Figure 4A, 4C).

According to HE-staining results, hippocampal pyramidal cells in Group C displayed normal morphologies with spherical pale-stained nuclei and uniformly stained cytoplasm. However, acidophilic changes and disordered neuronal arrangements were evident in the hippocampus of Group IR. After postconditioning with sevoflurane, these pathological changes in Group IR were ameliorated in Group IR+S, while the TLR4/MyD88/TRAF6 pathway agonist LPS partially reversed the protective effects of sevoflurane in Group A (Figure 4B).

### Sevoflurane postconditioning reduced neuroinflammation

**Figure 5:**
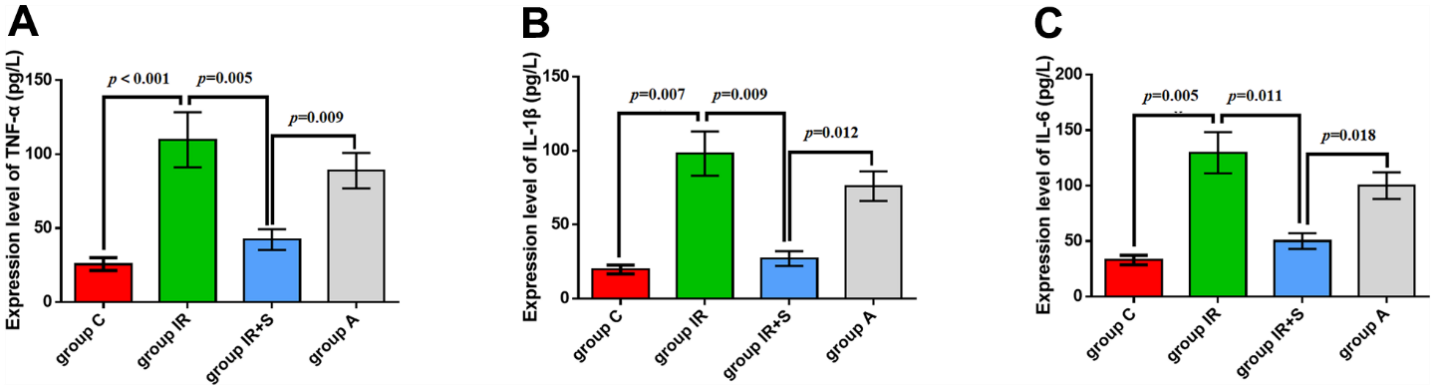
**Sevoflurane postconditioning reduced neuroinflammation.** mRNA levels of TNF-α (**A**), IL-1β (**B**), and IL-6 (**C**). Data are expressed as mean ± SD (n = 5 per group).

To explore how sevoflurane postconditioning prevented cerebral I/R injury, we evaluated levels of pro-inflammatory cytokines in rat hippocampi. As shown in Figure 5A–5C, TNF-α (*p* < 0.001), IL-1β (*p* = 0.007), and IL-6 (*p* = 0.005) levels in Group IR were increased compared with Group C; thus, cerebral I/R followed by sevoflurane postconditioning decreased cerebral I/R-induced increases in TNF-α (*p* = 0.005), IL-1β (*p* = 0.009), and IL-6 (*p* = 0.011). Furthermore, LPS partially reversed the protective effect of sevoflurane postconditioning in Group A[TNF-α (*p* = 0.009), IL-1β (*p* = 0.012), and IL-6 (*p* = 0.018)].

### Sevoflurane postconditioning inhibited the TLR4/MyD88/TRAF6 signaling pathway to exert a neuroprotective effect in cerebral I/R rats

**Figure 6:**
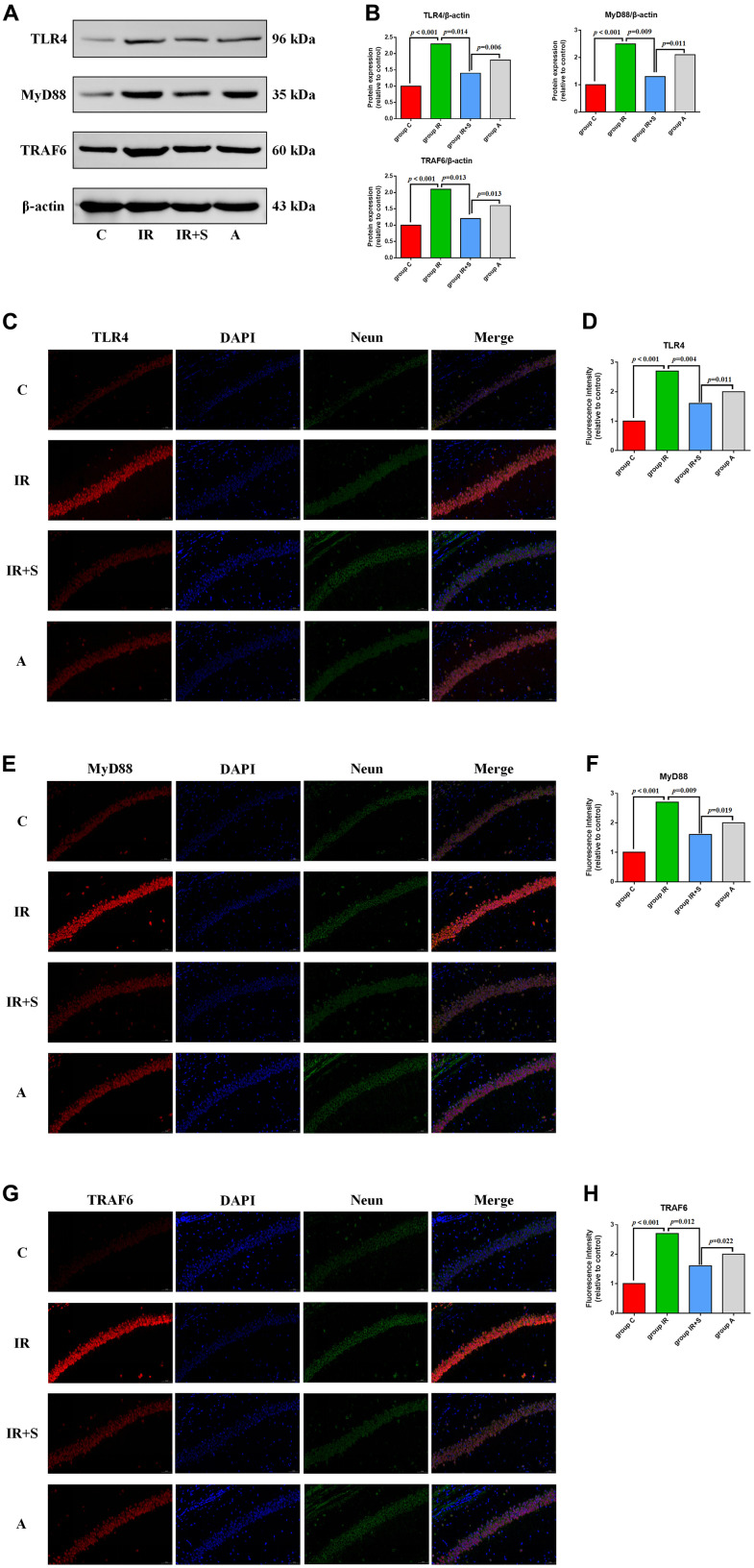
**Sevoflurane postconditioning inhibits the TLR4/MyD88/TRAF6 signaling pathway to exert a neuroprotective effect in cerebral I/R rats.** (**A**) Representative western blot of TLR4, MyD88, and TRAF6. (**B**) Representative photomicrographs of TLR4/DAPI staining (TLR4 in red and DAPI in blue), scale bar = 10 μm. (**C**) Representative photomicrographs of MyD88/DAPI staining (MyD88 in red and DAPI in blue), scale bar = 10 μm. (**D**) Representative photomicrographs of TRAF6/DAPI staining (TRAF6 in red and DAPI in blue), scale bar = 10 μm. (**E**) Percentages of TLR4/DAPI-positive cells. (**F**) Percentages of MyD88/DAPI-positive cells. (**G**) Percentages of TRAF6/DAPI-positive cells. (**H**) Percentages of TRAF6/DAPI-positive cells.

Previous research demonstrated that inhibition of the TLR4/MyD88/TRAF6 signaling pathway plays an important role in ameliorating cerebral I/R injury by several neuroprotective measures [23, 32, 33]. Therefore, we speculated that it also plays an important role in the alleviating effect of sevoflurane postconditioning in cerebral I/R rats. To test this hypothesis, we first evaluated expression levels of TLR4, MyD88, and TRAF by western blot analysis. TLR4 (*p* < 0.001), MyD88 (*p* < 0.001), and TRAF6 (*p* < 0.001) expression were significantly higher in the hippocampus of rats in Group IR compared with Group C. Sevoflurane postconditioning significantly downregulated the expression of TLR4 (*p* = 0.014), MyD88 (*p* = 0.009), and TRAF6 (*p* = 0.013) in Group IR+S while LPS partially reversed the protective effect of sevoflurane postconditioning in Group A[TLR4 (*p* = 0.006), MyD88 (*p* = 0.011), and TRAF6 (*p* = 0.013)] (Figure 6A and Supplementary Figure 1).

Immunofluorescence assay results show that the optical density of TLR4 (*p* < 0.001), MyD88 (*p* < 0.001), and TRAF6 (*p* < 0.001) in the hippocampus was visibly higher in Group IR compared with Group C. In Group IR+S, the density of TLR4, MyD88, and TRAF6 was increased after sevoflurane postconditioning [TLR4 (*p* = 0.004), MyD88 (*p* = 0.009), and TRAF6 (*p* = 0.012)], and this protective effect of sevoflurane was partly reversed by LPS in Group A[TLR4 (*p* = 0.011), MyD88 (*p* = 0.019), and TRAF6 (*p* = 0.022)] (Figure 6B–6G).

## DISCUSSION

**Figure 7:**
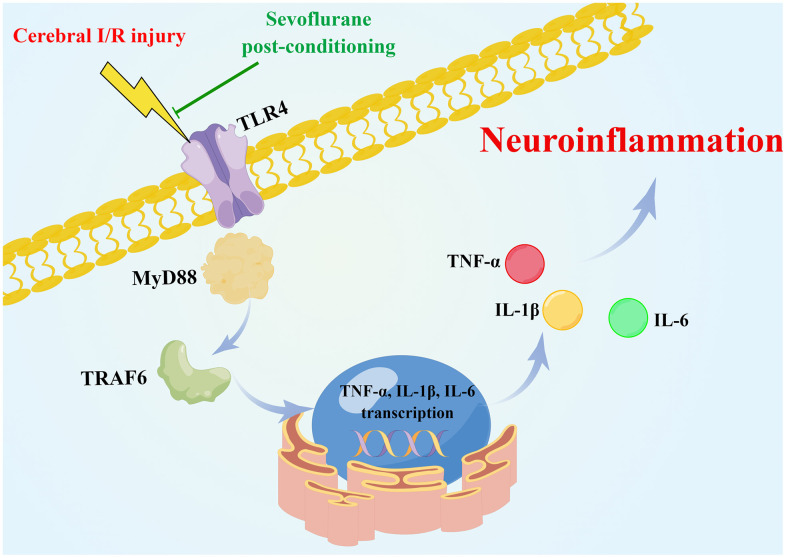
**Schematic of the neuroprotective effect of sevoflurane postconditioning (Drawn by Figdraw platform, ID:OATYA5cb8f).** Sevoflurane postconditioning ameliorates cerebral I/R injury in rats by inhibiting the TLR4/MyD88/TRAF6 signaling pathway to suppress neuroinflammatory responses.

Globally, there is an increased risk of death and disability associated with ischemic cerebrovascular disease. Despite the effectiveness of drugs for treating cerebral ischemia, further study of the pathophysiological and treatment mechanisms is still needed to develop new clinical treatment approaches. In the present study, we provide new mechanistic insights into how sevoflurane postconditioning ameliorates cerebral I/R injuries. As a result of bioinformatics analysis, 103 DEGs were identified, for which Toll-like receptor signaling was found to be significantly enriched pathway. Postconditioning with sevoflurane alleviated cerebral I/R injury by inhibiting the TLR4/MyD88/TRAF6 signaling pathway to suppress neuroinflammatory responses (Figure 7).

As a result of the unacceptably high incidence rate and mortality of cerebral I/R, it poses a heavy burden on patients, family members, and society. Many factors can cause cerebral I/R injury, such as calcium overload, inflammatory reactions, and free radicals. Among them, the inflammatory reaction is thought to play a major role in cerebral I/R injury occurrence and development. During cerebral I/R, numerous inflammatory factors are produced that further stimulate the body to produce more leukocytes. Moreover, in response to these inflammatory factors, leukocytes adhere to and damage endothelial cells, which release free radicals and vasoconstrictor factors that induce tissue damage [34]. Large numbers of infiltrating inflammatory cells can block cerebral microvessels, causing hypoperfusion of blood into the brain and worsening brain damage [35]. MCAO is a mature model of cerebral I/R injury that can accurately and successfully simulate the pathophysiological process of ischemic stroke. Thus, we developed a cerebral I/R model using MCAO [36]. As in She Y’s previous study [37], we prepared the cerebral I/R model with MCAO for 2 h, followed by reperfusion for 24 h. In agreement with previous studies, neurological scores and inflammation volumes were significantly increased after 24 h of reperfusion [37].

Scholars have explored many ways to prevent cerebral I/R injury. Wang showed that in hyperglycemia, oxymatrine may alleviate cerebral I/R injury by protecting microvessels [38]. In Chen’s study, clonidine protected against cerebral I/R injury by regulating protein expression of GluN3 subunits of the N-methyl-D-aspartate receptor [39]. Mao and colleagues showed that ligustilide reduced neuronal damage from I/R by encouraging mitophagy via PTEN-induced kinase 1/Parkin [40]. However, scholars have never stopped exploring protective measures against cerebral I/R injury. Sevoflurane, a widely used new inhalation anesthetic, is increasingly used for the study of anesthetic treatment of ischemic stroke in clinical anesthesia. However, exposure to sevoflurane at different doses and for different periods of time may induce different effects. Several studies showed repeated exposure or high dose and high concentration exposure combined with surgical operation may cause neurotoxicity and cognitive impairment in neonates or elderly patients, especially combined with surgery [41–43]. While a large number of studies have found the neuroprotective effects sevoflurane in POCD model and CIR model. Animal studies demonstrated a neuroprotective effect of sevoflurane on cerebral I/R injury, including pre- and postconditioning with sevoflurane. According to Yang’s study, the apoptotic rate of neurons in rats was reduced by sevoflurane through its effect on the E2F transcription factor 1/enhancer of zeste homolog 2/tissue inhibitor of metalloproteinases 2 regulatory axis, thus protecting rats from cerebral I/R injury [44]. In addition, multiple rounds of sevoflurane preconditioning were shown to protect against cerebral I/R injury by activating the endogenous antioxidant response through Kelch-like ECH-associated protein 1 downregulation-dependent nuclear factor-E2-related factor 2 [45]. Postconditioning with sevoflurane decreased cerebral I/R injury by increasing Nrf2/heme oxygenase 1 expression via protein kinase C signaling [46]. It is relatively easy and practical to use sevoflurane postconditioning as a brain protection measure in clinical practice. Therefore, referring to the research method of Hwang [26], we adopted the postconditioning method of inhalation of 2.5 vol% sevoflurane for 10 min followed by a washout period of 10 min after ischemia in the current study. The results showed the cognitive function improved significantly after sevoflurane postconditioning which is Consistent with Hwang's research results [26].

Neuroinflammatory reaction triggered by multiple cytokines plays an important role in the process of cerebral I/R injury. Proinflammatory cytokines, including TNF-α, IL-6 and IL-1β, have been detected to be responsible for the extension of the cerebral I/R zone in animal models. TNF-α is a pro-inflammatory response factor produced by monocyte macrophages and secreted and released by activated microglia. It not only can harm the neurological system and exert neurotoxic effects, such as neuronal cell death and neurovascular edema, it can cause the body to begin an inflammatory response [47, 48]. When brain tissue is injured by cerebral I/R, TNF-α production and secretion increases rapidly. In the presence of rapidly increased TNF-α, nerve cells can become damaged, their membranes can be hydrolyzed, and the structure of nerve cells can be destroyed [49]. Moreover, TNF-α can promote inflammatory cells to aggregate in the cerebral ischemic area, releasing vasoactive substances that damage the blood-brain barrier [50]. IL-6 is a precursor secreted by activated T cells and fibroblasts that produce B cells and antibodies. *In vivo*, TNF-α can enhance the lysis function of natural killer cells when combined with colony-stimulating factors [51]. IL-1β, an important member of the IL-1 family, participates in anti-inflammatory functions, as well as cell proliferation, differentiation, and apoptosis, as a key pro-inflammatory cytokine [52]. The decrease in the level of TNF-α, IL-6, and IL-1β after sevoflurane postconditioning in cerebral I/R model identified in the present study demonstrates that there is an closely association between TNF-α, IL-6, and IL-1β and cerebral I/R injury which is similar to the results of Hwang’s study [26].

Many mechanisms contribute to cerebral I/R injury, and inflammation is one of the most important. TLRs are believed to be the only transmembrane proteins that transmit extracellular antigen recognition information to cells and trigger inflammatory reactions in mammals. TLR receptors are known as pattern recognition receptors because they recognize pathogens and initiate innate immunity at an early stage of pathogen invasion [53]. TLR4 is mainly composed of three regions: intracellular, extracellular and transmembrane. The extracellular regions with large sequence differences can enable the body to carry out immune defense response. Studies have shown that TLR4 is expressed in hippocampus and participates in the pathogenesis of cerebral I/R injury [54, 55]. The MyD88 A is an important connecting molecule in TLR receptor signal pathway, and plays an important role in the occurrence and development of diseases. TLR4 recruits MyD88, binds to TRAF6, and eventually transmits information. Based on the results of bioinformatic analysis, “Toll-like receptor signaling pathway” is one of the top five enriched pathways in the cerebral I/R injury model. As a typical path in Toll-like receptor signaling pathway, in the current study, we investigated the protective effect of sevoflurane postconditioning in cerebral I/R rats and further explore the role of TLR4/MyD88/TRAF6 signaling pathway in it. Evidence suggests that cerebral I/R can cause brain injury by activating the TLR4/MyD88/TRAF6 pathway and increasing neuroinflammatory responses [56]. According to the present findings, sevoflurane postconditioning inhibited activation of the TLR4/MyD88/TRAF6 pathway, reduced neuroinflammatory responses, and relieved neurological symptoms; moreover, this protective effect was partially reversed by TLR4 agonists.

This study has certain limitations. First, in the experiment, we only used 2 vol% sevoflurane for postconditioning and did not explore whether there was a dose-dependent neuroprotective effect of postconditioning with different sevoflurane concentrations. Second, while the inflammatory cascade can occur within a few days after cerebral I/R injury, our study only explored pathological changes and activation of the TLR4/MyD88/TRAF6 pathway 24 h after reperfusion. Finally, neuronal injury following brain I/R injury has been linked to TLR2 in previous studies. In this study, we only discussed the neuroinflammatory response mediated by TLR4. Thus, additional research is needed on the role of other TLR family members in neuroprotection elicited by sevoflurane postconditioning.

## CONCLUSIONS

The results of our study show that sevoflurane postconditioning ameliorated cerebral I/R injury in rats and inhibited the TLR4/MyD88/TRAF6 signaling pathway to suppress neuroinflammatory responses involved in the underlying neuroprotective mechanisms. As a result, new strategies for detecting and treating cerebral I/R injury may be developed to improve I/R outcomes.

## Supplementary Material

Supplementary Figures

Supplementary Table 1

Supplementary Table 2

Supplementary Table 3
