# Supplementary material for: Sevoflurane postconditioning ameliorates cerebral ischemia-reperfusion injury in rats via TLR4/MyD88/TRAF6 signaling pathway
Source: Aging (Albany NY). 2022 Dec 29;14(24):10153–70. doi: 10.18632/aging.204461 (PMC9831726; doi:10.18632/aging.204461)
Supplement: Supplementary Figures [file aging-14-204461-s001.pdf]

SUPPLEMENTARY FIGURES

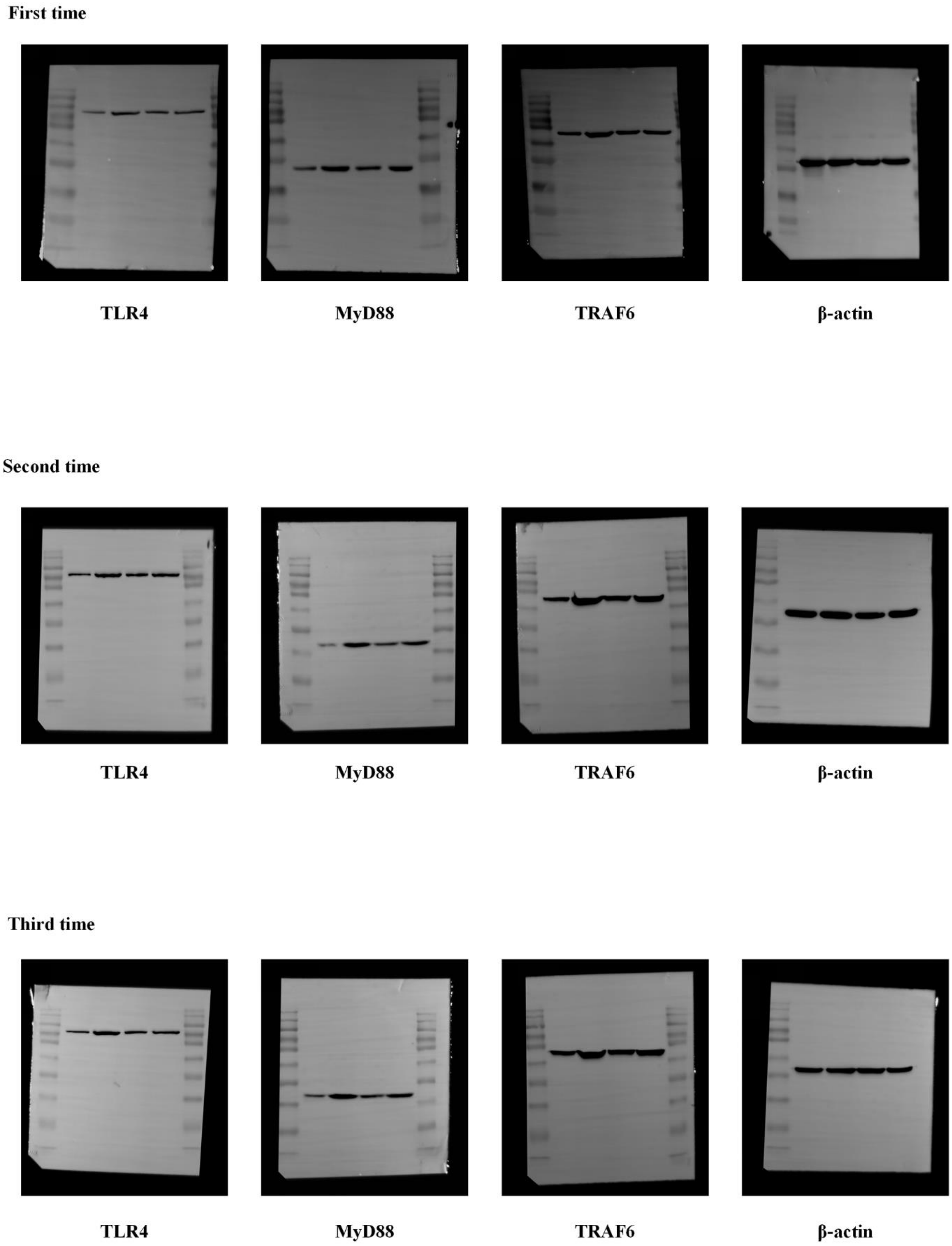

Supplementary Figure 1. Original stripe of Western blot.

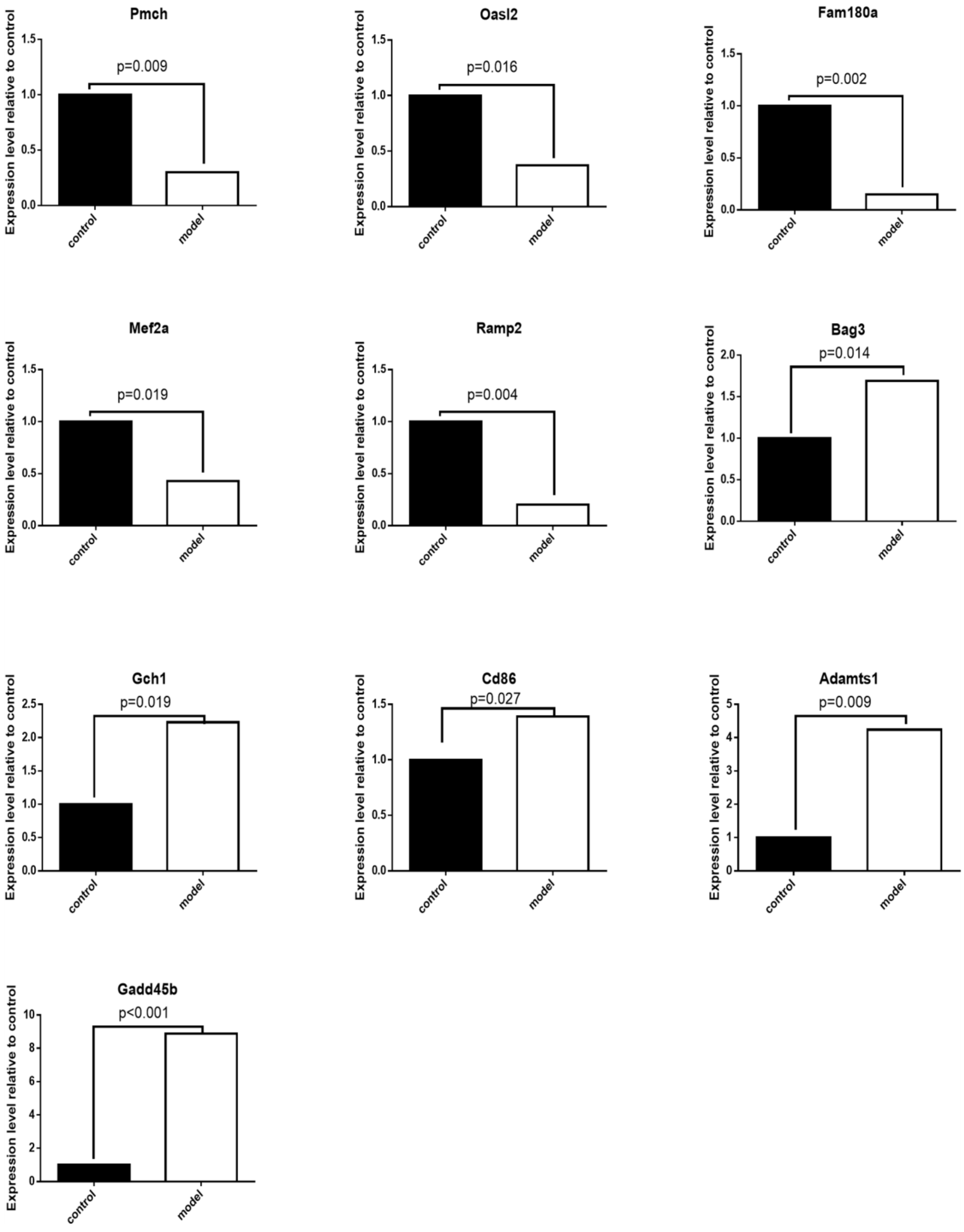

Supplementary Figure 2. Representative RT-PCR results of differential gene.
